# Brightness Discrimination in the Crepuscular Moth *Grapholita molesta* (Busck, 1916) Under Dappled Light

**DOI:** 10.3390/insects17060558

**Published:** 2026-05-28

**Authors:** Xiaofan Yang, Bao Li, Tongtong Huang, Guoshu Wei, Yafei Ge, Yanran Wan

**Affiliations:** 1 IPM Innovation Center of Hebei Province, Plant Protection Institute, Hebei Academy of Agriculture and Forestry Sciences, Baoding 071000, China; yangxiaofan87@126.com; 2 College of Plant Protection, Hebei Agricultural University, Baoding 071000, China; yangxiaofan19@163.com (B.L.); weiguoshu03@aliyun.com (G.W.); 3 Pingshan County Agriculture and Rural Affairs Bureau, Pingshan 050400, China; hxlhjfhtt0521@163.com

**Keywords:** *Grapholita molesta*, crepuscular moth, brightness discrimination, dappled light, oviposition preference

## Abstract

The light environment in orchards is highly heterogeneous, characterized by dappled light caused by sunlight filtering through plant canopies, and poses a challenge for the color perception of insects. For the crepuscular moth *Grapholita molesta*, a global pest of fruit trees, females can visually discriminate between plants to locate young leaves with higher brightness or intensity for oviposition around dusk. However, whether dappled light affects this brightness discrimination performance remains unknown. In this study, we investigated the oviposition preference of *G. molesta* females between two brightness stimuli (light-green and dark-green), as well as among three different light intensities under uniform light, simulated dappled light, and complex dappled light conditions at three ecologically relevant illuminances (100, 1, and 0.01 lx). *G. molesta* females laid significantly more eggs on light-green than dark-green stimuli under all three light conditions at each illuminance. Similarly, females consistently preferred high-intensity over lower-intensity light areas across uniform, dappled, and complex dappled light conditions, with more than 66% oviposition frequency. Our findings show that the brightness and intensity discrimination ability of *G. molesta* is not affected by dappled light, which may enable moths to accurately locate suitable oviposition sites even when the orchard canopy creates a heterogeneous light distribution.

## Simple Summary

The crepuscular moth *Grapholita molesta* is a major pest of fruit trees, and the females lay eggs on young leaves with a higher brightness or intensity around dusk. The light environment in orchards often varies between daylight and leaf shade, creating a dappled light condition. It is not known whether such dappled light affects brightness discrimination ability. In this study, we tested the preferences of *G. molesta* females between light-green (brighter brightness) and dark-green (darker brightness) stimuli, and between high- and low-light-intensity areas under uniform light, simulated dappled light, and complex dappled light conditions at three ecologically relevant illuminances (100, 1, and 0.01 lx). The results show that *G. molesta* females consistently preferred brighter green and higher-intensity stimuli in all light conditions, and dappled light did not reduce their brightness discrimination. These findings indicate that *G. molesta* females can reliably use brightness cues to choose oviposition sites, even in the challenging, heterogeneous light environments commonly found in orchards.

## 1. Introduction

Insects use color information for numerous tasks, including evaluating ambient light for phototaxis or navigation, detecting hosts for feeding or oviposition, and recognizing mates and predators for reproduction or survival [1,2]. In color perception, brightness is the most fundamental characteristic of a visual stimulus, describing the achromatic aspect associated with the intensity of perceived reflected or emitted light [3,4,5]. Although studies of insect color perception have generally focused on hue selectivity (i.e., the spectral distribution of reflected or emitted light), positive responses to brightness cues have been explicitly observed, particularly in some flower-visiting insects. For example, the diurnal species *Eristalis tenax*, *Papilio xuthus* and *Macroglossum stellatarum* all preferred the brighter of two shades with the same hue [6,7,8]. Given their higher signal-to-noise ratio, brightness cues may be particularly important under specific conditions, such as at night when light intensity is low. Accordingly, it has been reported that the crepuscular *Manduca sexta* and nocturnal *Deilephila elpenor* species exhibit a strong preference for higher brightness stimuli [5,9,10,11,12].

Natural illumination can vary significantly over the course of the day, between shaded and sunlit areas within a scene, and across different habitats such as forests and open fields [13,14]; these changes make reliable color perception particularly challenging. The insect visual system must therefore have mechanisms to compensate for differences in hue and brightness, i.e., color constancy or contrast constancy, to ensure that color information remains stable under changing illumination conditions [15,16]. For example, bees, butterflies, and hawkmoths can recognize learned target hues with high accuracy across varying illuminations [16,17,18,19,20]. In *Drosophila*, the luminance-sensitive pathway via the L3 neuron serves as a corrective signal that ensures contrast constancy, enabling accurate visual processing under rapidly changing light conditions [21]. Similarly, the bumblebee *Bombus terrestris* exhibits approximate color constancy and can discriminate between two similar hues differing in brightness (mauve and purple), although its accuracy is approximately 10% lower under simulated leaf shade than under daylight illumination [22].

The crepuscular oriental fruit moth, *Grapholita molesta* (Busck) (Lepidoptera: Tortricidae), is a major fruit-boring pest worldwide, causing significant economic damage to peach (*Prunus persica* L.), pear (*Pyrus communis* L.), apple (*Malus domestica* Borkh.), and other Rosaceae trees [23,24]. Gravid females prefer to lay eggs on the young leaves at the top of host plants around dusk [25,26]. While olfactory cues, specifically host volatiles, have been extensively documented as long-range orientation signals for distinguishing between host and non-host plants or among host species [27,28,29,30,31], visual cues become more important for perception in microhabitats. Our recent studies have shown that *G. molesta* females can visually discriminate oviposition sites in microhabitats even under dim light, with a preference for higher brightness and light intensity [26,32]. The light environment in orchards is heterogeneous; in particular, dappled light, which results from sunlight filtering through the branches and leaves of the plant canopy, contributes to this variability for much of the growing season. However, it remains unclear whether such dappled light environments affect brightness or intensity discrimination performance.

In this study, we investigated the oviposition preferences of *G. molesta* females between two brightness stimuli (light-green and dark-green) under simulated uniform, dappled, and complex dappled light conditions at three ecologically relevant illuminances (100, 1, and 0.01 lx, corresponding to twilight, late twilight, and moonlight). We also compared the preference for higher versus lower light intensities presented in choice tests.

## 2. Materials and Methods

### 2.1. Insects

A colony of *G. molesta* was established from wild-caught larvae in a peach orchard in Shunping, Hebei, China in August 2020. The colony was maintained in artificial climate chambers at 26 ± 1 °C, 70 ± 5% relative humidity (RH) under a 15 h:9 h light:dark cycle. Larvae were mass-reared on apples until they reached the third instar and were then transferred to an artificial diet [33] and adults were fed a 10% honey solution on cotton balls. Newly emerged adults were sexed and individually housed in glass tubes (1.8 cm diameter, 8 cm height) without access to food or water. Groups of fifteen 2-day-old females and males were paired 3–4 h before the onset of the scotophase. During the ensuing hours, copulating pairs were removed gently and separated 12 h later. Mated 3-day-old females were collected for oviposition preference experiments.

### 2.2. Experimental Setup and Stimuli

**Figure 1:**
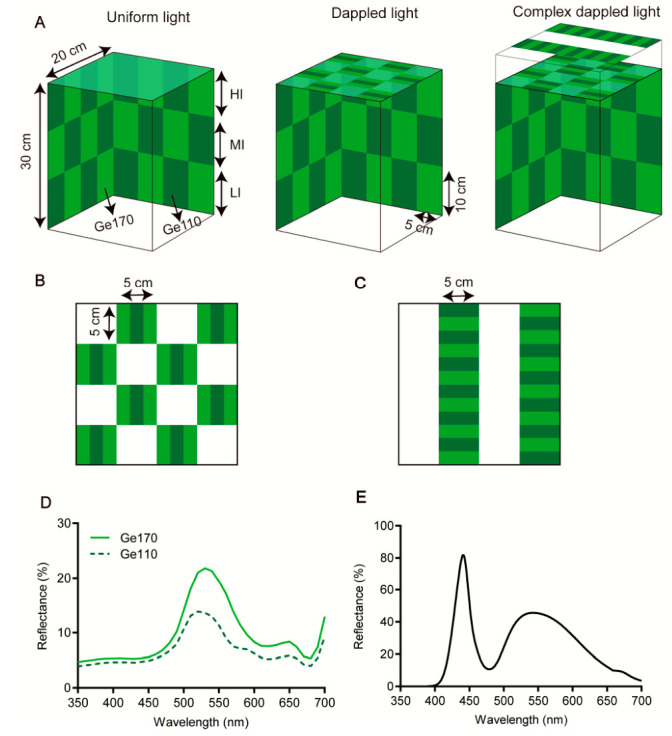
Experimental setup and stimuli. (**A**) Schematic diagram of the experimental boxes used to test the oviposition preference of *G. molesta*. A white LED lamp was positioned 50 cm above the box, and light-green (Ge170) and dark-green (Ge110) colored stimuli were presented alternately on the box wall. (**B**) Pattern of simulated dappled light. Light-green and dark-green patches represent young and old leaf-shade light filters, while white patches indicate areas illuminated by LEDs. (**C**) The second layer of the complex dappled light pattern. (**D**) Reflectance spectra of colored papers. (**E**) Emission spectra of the illuminating white LEDs.

Experiments were conducted in a cuboid cardboard box (20 × 20 × 30 cm, Figure 1A) in a dark room at 26 ± 1 °C, 60% ± 5% RH. The floor of the box was covered by rough white paper to prevent undesired oviposition. The stimuli, hereafter called light-green (Ge170) and dark-green (Ge110), were represented by twelve rectangular papers (5 × 10 cm) arranged vertically in an alternating 4 × 3 grid on each side of the box, simulating young and old peach leaves, respectively. The light-green and dark-green rectangles were printed on white matte paper (100 g/m^2^) using an inkjet printer (L801, Seiko Epson Corp., Nagano, Japan) with custom RGB settings following Yang et al. (2020) [32]; their reflectance spectra (Figure 1D) show that they shared the same chromatic properties but differed in brightness (achromatic properties). Each paper was laminated with anti-reflection film to provide a smooth thigmotactic surface while preventing contamination from moth excreta. We simulated leaf-shade conditions with light-green and dark-green filters placed above the box. These filters were produced by printing on tracing paper (0.05 mm thick) with same RGB settings as those used for the paper rectangles.

The experimental setup was illuminated from above (>50 cm) by a white LED lamp (18 W; Guangzhou Xinyuan Optoelectronic Technology Co., Ltd., Guangzhou, China). Corresponding to the light intensity which decreased with distance from the light source, the setup was vertically divided from top to bottom into three light-intensity areas without any physical barriers: a higher-intensity zone (top 1/3), a mid-intensity zone (middle 1/3), and a lower-intensity zone (bottom 1/3). The illumination was broad-spectrum with two emission peaks in the blue and green–yellow region (Figure 1E). Experiments were performed under three ecologically relevant illuminances: 100 lx (twilight), 1 lx (late twilight) and 0.01 lx (moonlight). Changes in illuminance do not affect the spectral distribution of LED-emitted light. Light intensity was measured at the top center of the experimental boxes using a radiometer (IL1700, International Light Research, Peabody, MA, USA) and adjusted by neutral density filters (Melles Griot, Rochester, NY, USA).

### 2.3. Experimental Procedure

To investigate whether dappled light affects brightness and intensity discrimination in *G. molesta*, we conducted three sets of experiments under illuminations of 100, 1 and 0.01 lx. First, oviposition preference was assessed under uniform lighting conditions. A large sheet of PVC plastic wrap was placed above the experimental box to ensure uniform illumination from the LEDs. Second, we created a dappled light environment by placing a leaf-shade filter above the box. The filter was arranged in a 4 × 4 alternating checkerboard patterns, consisting of eight colored squares (5 × 5 cm) and eight clear PVC-wrap squares (5 × 5 cm) (Figure 1B). This design allowed half of the box to be illuminated through the colored filters and the other half directly by LEDs, simulating the patchy light penetrating a leaf canopy. The colored squares were composed of two light-green and one dark-green patch [22]. Third, to introduce a more complex dappled light environment, we overlaid a second filter onto the checkerboard filter used in the second experiment. This additional filter was composed of alternating vertical stripes: two columns of light-green interleaved with dark-green stripes, and two columns of clear PVC wrap (Figure 1C). Each experiment was repeated 10 times.

All experiments began at 17:00, 3 h before the onset of the scotophase (when the moths start to oviposit). Ten 3-day-old mated females were introduced into the experimental boxes and allowed to lay eggs for 15 h overnight. The following morning, the females were removed, and the number of eggs laid on light-green and dark-green rectangles were recorded. When presenting stimuli to the moths, the anti-reflection laminating film was carefully cleaned with 75% alcohol. Females were tested only once.

### 2.4. Statistical Analysis

We used the Wilcoxon signed-rank test to compare the preference of *G. molesta* females between light-green and dark-green colors under different illuminations. Generalized linear models (GLMs) and Bonferroni post hoc tests were used to compare the preference across different light intensities, and to assess the differences in response to green brightness and light intensity under uniform light, dappled light and complex dappled light conditions. Females with a fecundity of fewer than 10 eggs were recorded but excluded from the statistical analysis. All statistical analyses were conducted using SPSS 27.0 (SPSS Inc., Chicago, IL, USA).

## 3. Results

### 3.1. Oviposition Responses to Green Brightness

**Figure 2:**
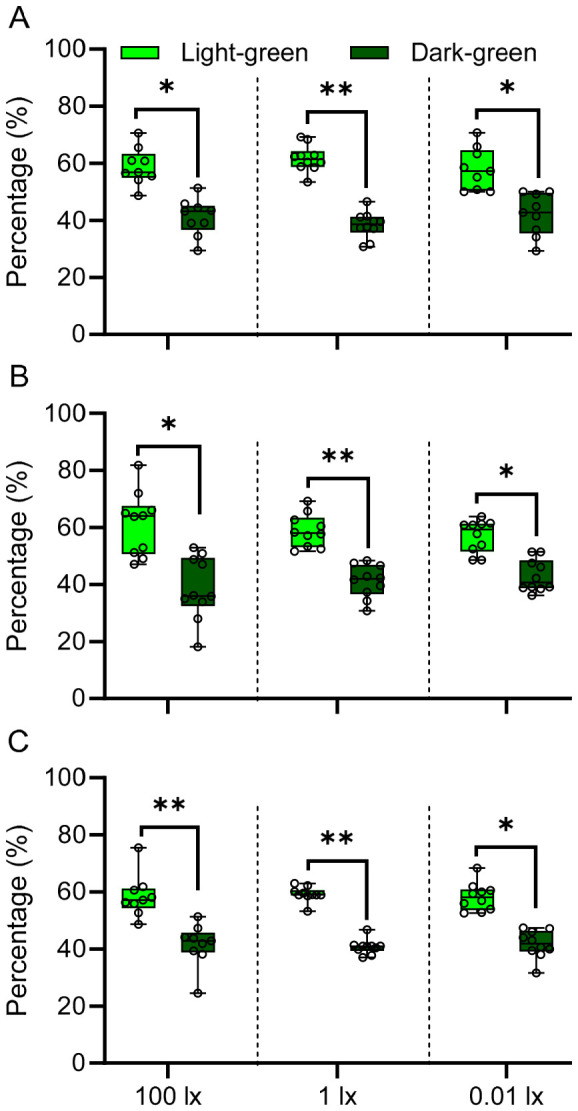
Oviposition preference of *G. molesta* in choices between light-green (Ge170) and dark-green (Ge110) colors under uniform light (**A**), dappled light (**B**) and complex dappled light (**C**). Data shown as mean ± SE, n = 10 experiments, 100 moths. Asterisks indicate significant differences (Wilcoxon signed-rank test; * *p* < 0.05; ** *p* < 0.01).

**Figure 3:**
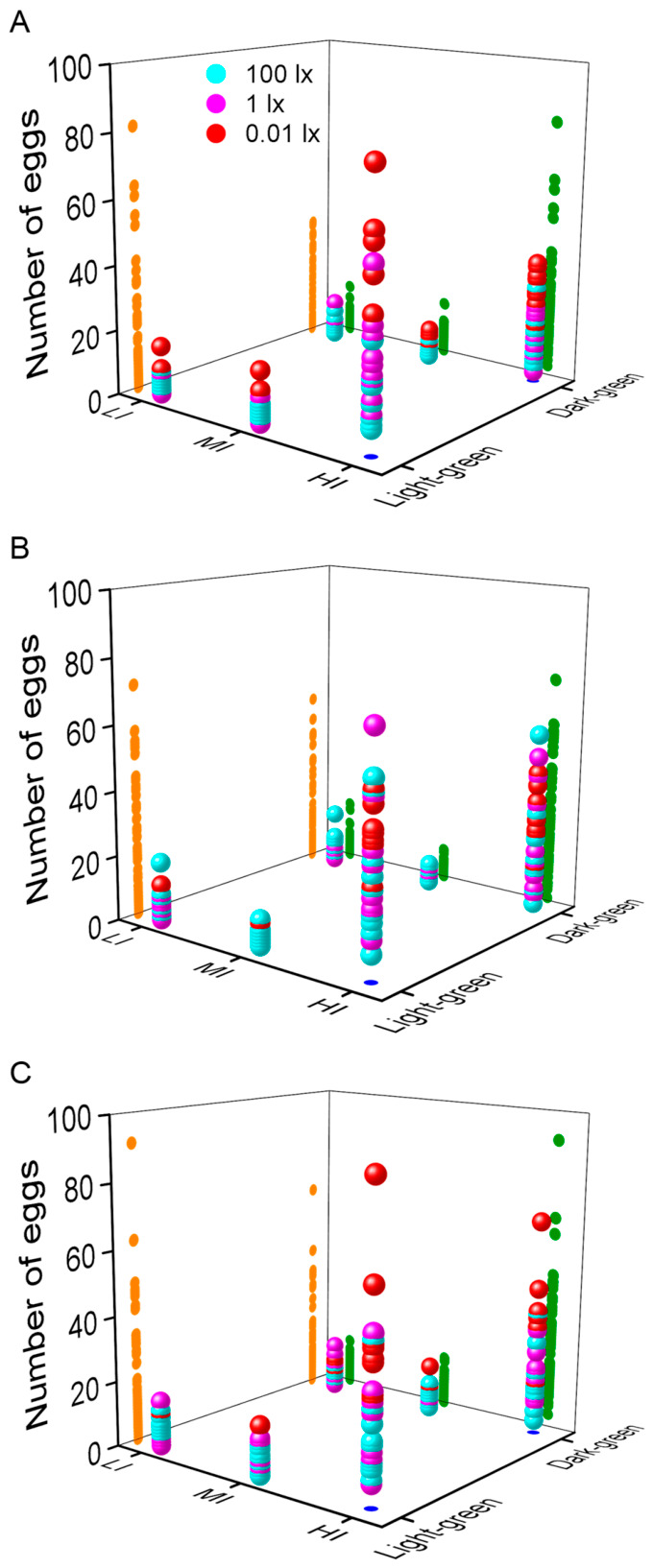
Number of eggs laid by *G. molesta* females on light-green and dark-green rectangles in higher-intensity (HI), mid-intensity (MI) and lower-intensity (LI) zones under illuminances of 100, 1 and 0.01 lx. (**A**–**C**) Oviposition preferences were tested under uniform light (**A**), dappled light (**B**) and complex dappled light (**C**). Each point represents the number of eggs laid by a single female. Orange, deep blue and green represent the mapping of each point onto the X-, Y-, and Z-axes, respectively.

*G. molesta* females exhibited a similar oviposition response to green brightness among uniform light, dappled light and complex dappled light conditions (Table 1, *p* > 0.05). Under all three light conditions, females significantly preferred to lay eggs on the light-green (Ge170) over the dark-green (Ge110) paper at illuminances of 100, 1 and 0.01 lx, with more than a 57% oviposition frequency (Figure 2 and Figure 3) (uniform light: Z_100 lx_ = −2.547, Z_1 lx_ = −2.803, Z_0.01 lx_ = −2.366; dappled light: Z_100 lx_ = −2.346, Z_1 lx_ = −2.803, Z_0.01 lx_ = −2.497; complex dappled light: Z_100 lx_ = −2.547, Z_1 lx_ = −2.805, Z_0.01 lx_ = −2.803, all *p* < 0.05).

### 3.2. Oviposition Responses to Light Intensity

**Figure 4:**
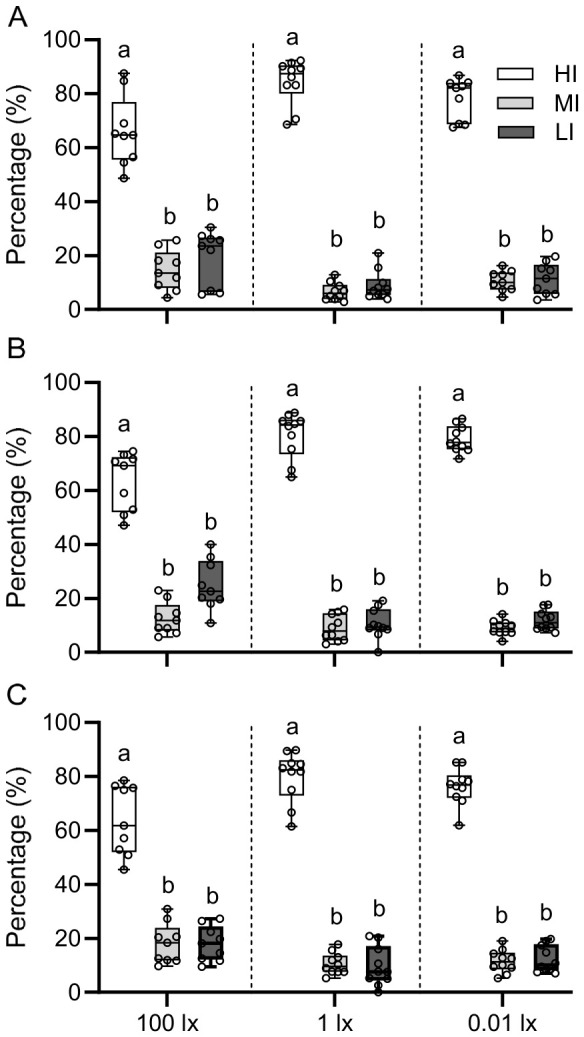
Oviposition preference of *G. molesta* in choices among higher-intensity (HI), mid-intensity (MI) and lower-intensity (LI) conditions under uniform light (**A**), dappled light (**B**) and complex dappled light (**C**). Data shown as mean ± SE, n = 10 experiments, 100 moths. Different letters above bars indicate significant differences at the *p* < 0.05 level (GLM followed by Bonferroni test).

Under uniform light, *G. molesta* females consistently exhibited a strong preference to lay eggs in the higher-intensity (HI) areas, with oviposition frequencies of 66.16%, 85.39% and 78.10% at illuminances of 100, 1 and 0.01 lx, respectively, and fewer eggs were laid in mid-intensity (MI) and lower-intensity (LI) areas (100 lx: F = 67.482, *p* < 0.001; 1 lx: F = 455.549, *p* < 0.001; 0.01 lx: F = 375.394, *p* < 0.001). Similarly, females significantly preferred HI areas under both dappled light and complex dappled light across all tested illuminances (Figure 3 and Figure 4, *p* < 0.001). These results suggest that the intensity discrimination of *G. molesta* is not affected by dappled light (Table 1, *p* > 0.05).

## 4. Discussion

As a moth moves through orchards in search of leaves or fruits for oviposition, it experiences different illuminations, from bright daylight to patchy leaf shade. Here, we investigated whether such dappled light environments affect brightness discrimination in the crepuscular moth *G. molesta*. Our results demonstrate that *G. molesta* females consistently preferred light-green over dark-green stimuli, and higher-intensity over lower-intensity light areas, across uniform, dappled, and complex dappled light conditions under the ecologically relevant illuminances tested (100, 1, and 0.01 lx). These findings suggest that brightness and intensity discrimination in *G. molesta* remains stable across heterogeneous light environments, and so they may be able to recognize oviposition sites with high accuracy in orchards where dappled light prevails for much of the growing season. To distinguish differences in brightness or light intensity under changing illumination conditions, *G. molesta* requires a highly sensitive visual system. Indeed, this crepuscular moth possesses superposition compound eyes, which are characterized by high light capture ability due to the presence of a clear zone separating crystalline cones from the rhabdom layer [34]. This structural adaptation improves visual reliability. Potentially, this reliability can be further improved by neural summation of light in space and time, which substantially increases contrast sensitivity and visual information rate, thereby supporting stable visual processing under rapidly changing light conditions [35].

Color or contrast constancy, the ability to perceive hue or brightness as stable under changing illumination, requires the visual system to compensate for illumination changes [15,16,20]. Here, the failure of dappled light to alter oviposition preferences for brightness and intensity in *G. molesta* suggests that females may employ luminance-based contrast constancy mechanisms, similar to those described in *Drosophila* [21]. Briefly, luminance-based contrast constancy is the ability of an insect’s visual system to detect a potential plant or predator by the luminance of the object against the luminance of the background. Alternatively, the moths might have evolved effective visual adaptation mechanisms to integrate brightness information over space or time, averaging out local variations introduced by leaf-shade patterns. Such strategies would be adaptive for a crepuscular species active during periods of rapid and patchy light changes. Future studies could investigate the luminance processing pathways and retinal mechanisms of *G. molesta* to elucidate the mechanistic basis of its robust brightness discrimination under variable light.

In contrast to our findings, Arnold and Chittka (2012) reported that bumblebees *B. terrestris* showed reduced color discrimination accuracy under simulated leaf shade, particularly when distinguishing between similar hues that differed primarily in brightness [22]. This discrepancy may reflect differences in visual ecology: diurnal bees rely heavily on chromatic cues for flower discrimination, whereas crepuscular *G. molesta* may depend more on achromatic (brightness) cues, which are less affected by spectral shifts in shade and can be detected with a higher signal-to-noise ratio in dim light [5,36].

The preference for brighter and higher-intensity stimuli observed in *G. molesta* across all experimental conditions aligns with our previous findings that this species relies on brightness or intensity cues for oviposition in dim light [26,32] and is consistent with the visual behavior of other crepuscular and nocturnal lepidopterans. For example, the crepuscular *M. sexta* and nocturnal *D. elpenor* both show strong innate preferences for higher-intensity stimuli [5,10,11,12]. This study extends these findings by demonstrating that this preference persists even under dappled light, thus allowing *G. molesta* females to accurately locate oviposition sites even when the orchard canopy creates patchy light distribution. This adaptation may enhance offspring survival, as young leaves at the top of host plants provide optimal nutritional conditions for larval development [37].

Notably, our experimental females were socially naive, reared in isolation from eclosion to oviposition. Social isolation can alter insect oviposition behavior: isolated females of flies, cockroaches, and beetles often lay fewer eggs or show altered host preferences compared to grouped conspecifics [38,39,40]. Future studies should test socially experienced females to validate whether social cues modulate brightness-based oviposition choices in *G. molesta*. While our study isolated visual cues to test brightness discrimination, insects often use both visual and olfactory cues in nature. Under field conditions, brightness preferences likely interact with olfactory cues: a bright leaf may be rejected if it emits repellent volatiles, while a dim leaf may be accepted if it releases attractive volatiles. Our findings provide a foundational understanding of visual preference, but field studies integrating both visual and chemical cues are needed to fully explain oviposition site selection in *G. molesta*.

In conclusion, our study provides strong evidence that *G. molesta* females maintain reliable brightness discrimination across uniform, dappled, and complex dappled light environments, at illuminances ranging from twilight to moonlight. These findings advance our understanding of insect visual adaptation to variable light environments and may have implications for developing visual-based pest management strategies, such as optimizing the brightness contrast of traps or repellent surfaces. Future research into the mechanistic basis of brightness discrimination in *G. molesta* will further elucidate the evolutionary and physiological adaptations of crepuscular insect visual systems to low and heterogeneous light.

## Figures and Tables

**Table 1:** Influence of dappled light on brightness/intensity discrimination of *G. molesta*.

Light Intensity	Oviposition Preference Tests	F	*p*-Value
100 lx	Responses to green brightness	0.298	0.745
	Responses to light intensity	0.006	0.994
1 lx	Responses to green brightness	1.162	0.328
	Responses to light intensity	0.000	1.000
0.01 lx	Responses to green brightness	4.733	0.131
	Responses to light intensity	0.000	1.000

## Data Availability

The original contributions presented in this study are included in the article. Further inquiries can be directed to the corresponding authors.

## References

[1] Song B.M., Lee C.H. (2018). Toward a mechanistic understanding of color vision in insects. Front. Neural Circuits.

[2] van der Kooi C.J., Stavenga D.G., Arikawa K., Belušič G., Kelber A. (2021). Evolution of insect color vision: From spectral sensitivity to visual ecology. Annu. Rev. Entomol..

[3] Prokopy R.J., Owens E.D. (1983). Visual detection of plants by Herbivorous insects. Annu. Rev. Entomol..

[4] Christenson M.P., Sanz Diez A., Heath S.L., Saavedra-Weisenhaus M., Adachi A., Nern A., Abbott L.F., Behnia R. (2024). Hue selectivity from recurrent circuitry in *Drosophila*. Nat. Neurosci..

[5] van der Kooi C.J., Kelber A. (2022). Achromatic cues are important for flower visibility to hawkmoths and other insects. Front. Ecol. Evol..

[6] Kelber A. (2005). Alternative use of chromatic and achromatic cues in a hawkmoth. Proc. R. Soc. Lond. B Biol. Sci..

[7] Kinoshita M., Takahashi Y., Arikawa K. (2012). Simultaneous brightness contrast of foraging Papilio butterflies. Proc. R. Soc. Lond. B Biol. Sci..

[8] An L., Neimann A., Eberling E., Algora H., Brings S., Lunau K. (2018). The yellow specialist: Dronefly *Eristalis tenax* prefers different yellow colours for landing and proboscis extension. J. Exp. Biol..

[9] Kelber A., Balkenius A., Warrant E.J. (2002). Scotopic colour vision in nocturnal hawkmoths. Nature.

[10] Johnsen S., Kelber A., Warrant E., Sweeney A.M., Widder E.A., Lee R.L., Hernández-Andrés J. (2006). Crepuscular and nocturnal illumination and its effects on color perception by the nocturnal hawkmoth *Deilephila elpenor*. J. Exp. Biol..

[11] Goyret J., Pfaff M., Raguso R.A., Kelber A. (2008). Why do *Manduca sexta* feed from white flowers? Innate and learnt colour preferences in a hawkmoth. Naturwissenschaften.

[12] Kuenzinger W., Kelber A., Weesner J., Travis J., Raguso R.A., Goyret J. (2019). Innate colour preferences of a hawkmoth depend on visual context. Biol. Lett..

[13] Endler J.L. (1993). The colour of light in forests and its implications. Ecol. Monogr..

[14] Cronin T.W., Johnsen S., Marshall J., Warrant E.J. (2014). Visual Ecology.

[15] Fabian J., Nordström K., Ogawa Y. (2020). Insect vision: Novel mechanism for contrast constancy in dim light. Curr. Biol..

[16] Werner A. (2022). Understanding insect colour constancy. Philos. Trans. R. Soc. Lond. B Biol. Sci..

[17] Werner A., Menzel R., Wehrhahn C. (1988). Color constancy in the honeybee. J. Neurosci..

[18] Kinoshita M., Arikawa K. (2000). Colour constancy in the swallowtail butterfly *Papilio xuthus*. J. Exp. Biol..

[19] Dyer A.G., Chittka L. (2004). Biological significance of distinguishing between similar colours in spectrally variable illumination: Bumblebees (*Bombus terrestris*) as a case study. J. Comp. Physiol. A Neuroethol. Sens. Neural Behav. Physiol..

[20] Balkenius A., Kelber A. (2004). Colour constancy in diurnal and nocturnal hawkmoths. J. Exp. Biol..

[21] Ketkar M.D., Sporar K., Gür B., Ramos-Traslosheros G., Seifert M., Silies M. (2020). Luminance information is required for the accurate estimation of contrast in rapidly changing visual contexts. Curr. Biol..

[22] Arnold S.E., Chittka L. (2012). Illumination preference, illumination constancy and colour discrimination by bumblebees in an environment with patchy light. J. Exp. Biol..

[23] Lü D., Yan Z., Hu D., Zhao A., Wei S., Wang P., Yuan X., Li Y. (2022). RNA sequencing reveals the potential adaptation mechanism to different hosts of *Grapholita molesta*. Insects.

[24] Kirk H., Dorn S., Mazzi D. (2013). Worldwide population genetic structure of the oriental fruit moth (*Grapholita molesta*), a globally invasive pest. BMC Ecol..

[25] Myers C.T., Hull L.A., Krawczyk G. (2006). Effects of orchard host plants on the oviposition preference of the oriental fruit moth (Lepidoptera: Tortricidae). J. Econ. Entomol..

[26] Yang X.F., Lu Z.Y., Ran H.F., Ma A.H., Liu W.X., Li J.C., Wei G.S. (2022). Oviposition site selection in the crepuscular moth, *Grapholita molesta*: Does light matter?. Entomol. Exp. Appl..

[27] Chen L., Tian K., Xu X., Fang A., Cheng W., Wang G., Liu W., Wu J. (2020). Detecting host-plant volatiles with odorant receptors from *Grapholita molesta* (Busck) (Lepidoptera: Tortricidae). J. Agric. Food Chem..

[28] Il’Ichev A.L., Kugimiya S. (2009). Volatile compounds from young peach shoots attract males of oriental fruit moth in the field. J. Plant Interact..

[29] Najar-Rodriguez A., Orschel B., Dorn S. (2013). Season-long volatile emissions from peach and pear trees in situ, overlapping profiles, and olfactory attraction of an oligophagous fruit moth in the laboratory. J. Chem. Ecol..

[30] Natale D., Mattiacci L., Hern A., Pasqualini E., Dorn S. (2003). Response of female *Cydia molesta* (Lepidoptera: Tortricidae) to plant derived volatiles. Bull. Entomol. Res..

[31] Piñero J.C., Dorn S. (2009). Response of female oriental fruit moth to volatiles from apple and peach trees at three phenological stages. Entomol. Exp. Appl..

[32] Yang X.F., Li M.Y., Fan F., An L.N., Li J.C., Wei G.S. (2020). Brightness mediates oviposition in crepuscular moth, *Grapholita molesta*. J. Pest. Sci..

[33] Yang X.F., Fan F., Wang C., Wei G.S. (2016). Effect of host plants on the development, survivorship and reproduction of *Grapholita molesta* (Lepidoptera: Tortricidae) under laboratory conditions. Austral Entomol..

[34] Yang X.F., Ran H.F., Jiang Y.L., Lu Z.Y., Wei G.S., Li J.C. (2024). Fine structure of the compound eyes of the crepuscular moth *Grapholita molesta* (Busck 1916) (Lepidoptera: Tortricidae). Front. Physiol..

[35] Stöckl A.L., O’Carroll D.C., Warrant E.J. (2016). Neural summation in the hawkmoth visual system extends the limits of vision in dim Light. Curr. Biol..

[36] Osorio D., Vorobyev M. (2005). Photoreceptor sectral sensitivities in terrestrial animals: Adaptations for luminance and colour vision. Proc. R. Soc. Lond. B Biol. Sci..

[37] Myers C.T., Hull L.A., Krawczyk G. (2007). Effects of orchard host plants (apple and peach) on development of oriental fruit moth (Lepidoptera: Tortricidae). J. Econ. Entomol..

[38] Kawecki T.J. (1995). Adaptive plasticity of egg size in response to competition in the cowpea weevil, *Callosobruchus maculatus* (Coleoptera: Bruchidae). Oecologia.

[39] Fowler E.K., Leigh S., Rostant W.G., Thomas A., Bretman A., Chapman T. (2022). Memory of social experience affects female fecundity via perception of fly deposits. BMC Biol..

[40] Uzsák A., Schal C. (2012). Differential physiological responses of the German cockroach to social interactions during the ovarian cycle. J. Exp. Biol..

